# Adapting breastfeeding support in areas of socio-economic deprivation: a case study approach

**DOI:** 10.1186/s12939-021-01393-7

**Published:** 2021-03-20

**Authors:** Louise Hunt, Gill Thomson, Karen Whittaker, Fiona Dykes

**Affiliations:** 1 Maternal and Infant Nutrition and Nurture Unit (MAINN). School of Community Health and Midwifery, University of Central Lancashire (UCLan), Preston, PR12HE UK; 2 School of Nursing, UCLan, Preston, PR12HE UK

**Keywords:** Breastfeeding, Peer support, Service development, Socio-economic deprivation, Qualitative, Case study

## Abstract

**Background:**

There are inequalities in breastfeeding initiation and continuation rates, whereby socio-economically disadvantaged mothers are least likely to breastfeed. Breastfeeding peer support (BPS) interventions are recommended as a solution, and in the UK non-profit organisations are commissioned to deliver BPS services in areas of socio-economic deprivation. BPS interventions have a mixed evidence base, offering limited knowledge about the interaction between context and intervention and how this affects women’s experiences.

**Methods:**

This interpretive study used a case study methodology to explore how and why two BPS services developed their services in socio-economically deprived contexts. Methods aimed to generate holistic understanding of BPS service development. Data collected across both cases comprised; observation (*n* = 1), and semi-structured interviews with: mothers who had (*n* = 10) and had not (*n* = 9) engaged with the BPS services, peer supporters (PSs) (*n* = 9), community health professionals (*n* = 5), infant feeding co-ordinators (*n* = 2), non-profit organisation managers (*n* = 3), and public health commissioners (*n* = 2). Inductive grounded theory analytic techniques of open coding and constant comparisons, followed by cross case comparisons, were used to analyse the data.

**Results:**

The over-arching theme - *‘the transcending influence of society’* – offers insights into the underlying context and drivers impacting service development. It reflects how funding and data sharing arrangements determined service operation and the peer’s access to women. Four underpinning themes explain how: peer supporters were resourceful in adapting their services (‘*adapting and modifying the support’*); BPS organisations worked to enable women’s access to supportive breastfeeding environments, but did not necessarily focus service development on the needs of women living in areas of deprivation (‘*supporting women’s journeys to access’*); the BPS-professional connections for supporting access and how BPS could result in more supportive community environments (‘*embedding within healthcare practice’*); and how management practices precluded meaningful use of data to provide context led service development (‘*ways of using knowledge’*).

**Conclusions:**

Findings suggest that while PSs are commissioned to focus on those most in need, there is limited discussion, collection, or use of knowledge about women’s lives to develop needs-led service delivery. The key recommendation is the development of a social ecological tool to facilitate the use and application of contextual knowledge.

**Table:** 

This article is a part of the Interventions and policy approaches to promote equity in breastfeeding collection, guest-edited by Rafael Pérez-Escamilla, PhD and Mireya Vilar-Compte, PhD	

## Background

Despite clear worldwide evidence of the negative short and long-term health impacts for mothers and children of not breastfeeding [1], global rates remain below international targets [2]. In high income countries breastfeeding prevalence is higher among better educated, higher income families [1]. For example, in Australia breastfeeding initiation is 96%, with 39% of babies being exclusively breastfed for six months [3], however, 68% of babies from households classified in the lowest socio-economic banding were exclusively breastfed at one month of age, compared to 81% of those from the highest socio-economic banding [3]. In Canada exclusive breastfeeding until six months is associated with more years of maternal education [4], and in the USA 25.6% of babies are exclusively breastfed for six months [5], yet US mothers aged over 30 years, are more than twice as likely to breastfeed exclusively than those aged under 20 years [6]. In the UK 89% of mothers living in the least deprived areas initiated breastfeeding, compared to 73% of those living in the most deprived areas [7]. Overall, 81% of UK mothers initiated breastfeeding [7]. At 6 weeks 23% were breastfeeding exclusively and 55% giving some breastmilk, while at 6 months 1% of mothers were exclusively breastfeeding [7]. Systematic reviews have found additional support from both lay supporters and professionals positively affects breastfeeding outcomes [8, 9], and Breastfeeding Peer Support (BPS) interventions are nationally and internationally recommended to increase breastfeeding rates [10–12], and help address inequalities [11]. A recent survey of UK NHS organisations found that amongst those who responded, peer support was available in 56% of areas [13]. A quarter of the survey respondents also felt that peer support was not well accessed by mothers from poorer social backgrounds [13]. Peer support has been defined as:

The provision of emotional, appraisal, and informational assistance by a created social network member who possesses experiential knowledge of a specific behaviour or stressor and similar characteristics as the target population [14 , p. 329].

The BPS evidence base is mixed; qualitative research reveals that women value peer support in terms of the time it provides for breastfeeding support [15, 16], and it's promotion of maternal well-being, a sense of belonging and hope in women’s situations [16–18]. Qualitative research also reports BPS helps women to continue breastfeeding when they would otherwise have stopped e.g., [16, 19, 20, 21], although analysis of BPS trials in high-income countries (in particular the UK) have found them to be ineffective in increasing breastfeeding rates [22, 23]. Trial contexts vary however, and currently there is a lack of understanding about how interventions interact with and adapt to the context of service provision (i.e. social, cultural, economic, interpersonal issues).

Inequalities in health constitute a significant issue. Health inequalities are differences in health across a population which may be defined as ‘*systematic, socially produced (and therefore modifiable) and unfair’* ([24], p.2).. They follow a gradient so that a higher social position is associated with better health [25]. Evidence reviews have found differences in the conditions of daily life, or the social determinants of health, form ‘*a major part’* of the health inequalities found both within and between countries ([26], p.1).. Health services can have a positive impact on the social determinants of health by adopting proportionate universal policies that respond to local health needs and direct additional action and resource to communities where deprivation levels are higher [27].

The role of non-profit organisations in health policy is important to consider. Non-profit organisations seek to make money for a social purpose, or to provide a service people need [28]. Such organisational activity takes place in a space between the market, the state and the family, and can involve varied organisational types including faith and community groups, social enterprises and charities [29]. Current UK government policy envisages an important role for this sector within health services generally [30], and as part of efforts to impact health inequalities [27, 31]. The roots and membership of non-profit breastfeeding organisations arise from relatively wealthy women, traditionally referred to as ‘middle-class’ in the UK i.e., [32, 33], yet these organisations have responded to commissions to provide BPS interventions in areas of deprivation. Currently, there is little known about how these organisations adapt and develop services to meet the needs of the women they support. This study aimed to explore, and build theory about, how UK non-profit breastfeeding organisations developed BPS services for areas of deprivation. It was envisaged that such knowledge would benefit BPS providers and other health care professionals in the UK and elsewhere. Ultimately, women living in areas of deprivation would also benefit through shared good practice and recommendations.

## Methods

### Study design

This study employed case study methods. A case study is the study of the ‘real-life’ context of a defined system that is closely entwined with its setting [34]. This approach is beneficial when asking how/why questions arising from practice [34, 35], and for investigating process rather than outcomes [34]; a case study can accommodate a range of theoretical underpinnings and disciplinary perspectives [34–37]. We chose to adopt Stake’s [37] inductive approach that develops ideas into patterns to create theories [38], and recognises co-constructions between participants and researcher(s) [39]. This was because we adopted an interpretivist approach where interpretations are acknowledged to be influenced by researchers’ own perspectives [40]. The practice of reflexivity, whereby the influences and experiences, motives and agendas of researchers on both the participants and the research field are examined [36], was crucial throughout this study. The first author has a nursing background, has breastfed three children, and been involved in a paid and voluntary capacity with BPS projects run by a small non-profit organisation. She was unknown in the study areas and had no previous relationship with the non-profit organisations involved. The remaining authors have practice (FD, KW) and/or research (FD, KW, GT) experience in breastfeeding support. A reflexive interview designed to identify LH’s previous assumptions and values was undertaken with FD and KW before the study began, and a reflexive journal kept throughout, with ideas shared within the study team.

### BPS organisation and site selection

Although many small, locally arising organisations deliver non-professional BPS services, for the purposes of this study, interest lay in large national organisations commonly commissioned to run BPS in the UK. Choice of case study sites was guided by the opportunity to learn about how services had developed for the context [37], rather than as exemplars of best practice or to represent an organisation’s work. Practical considerations such as whether sites could provide opportunities to recruit adequate numbers of local women and the willingness of health service and peer support staff to be involved also guided site selection.

Organisation A is large and longstanding. Its BPS projects form one part of a suite of possible services and interventions to support parents in their transition to parenthood. Organisation B arose from a longer established organisation around twenty years ago. It aims to increase awareness about the value of breastfeeding to women, families, and society. It has a particular concern for women least likely to breastfeed and a long-term commitment to areas of deprivation. BPS projects are its main activity.

### Introduction to case study sites

Site 1 is an urban post-industrial part of Northern England comprising large areas of deprivation. An established black and minority ethnic community makes up 10–20% of the population. Most health services are UNICEF UK Baby Friendly Initiative accredited. In England, deprivation is measured by the index of multiple deprivation [41]. It ranks small areas from 1 (most deprived) to 32,844 (least deprived), and divides them into five portions or quintiles [41]. Quintile 1 areas form the most deprived portion [41]. In 2016, organisation A were commissioned to deliver universal postnatal peer support with a targeted element whereby mothers living in quintile 1 areas and young mothers under twenty would receive more of the resource. The hospital postnatal ward and neonatal unit hosted PSs, and all women discharged breastfeeding received a telephone phone call at 48 h. During the call, mothers were asked for their postcode allowing identification of those living in quintile 1 areas. Three PSs provided a proactive service, offering a home visit and ongoing text, phone, and home visit support as needed for six weeks, with an invitation to ongoing virtual resources and community groups. Women could also self-refer or be referred into the service by health professionals. The core service was provided by paid PSs, supplemented by volunteers.

Site 2 is an affluent area in Southern England with a small black and minority ethnic population and mix of urban and rural communities, each with pockets of deprivation. Health services are UNICEF UK Baby Friendly Initiative accredited. In 2017, organisation B was commissioned to provide a universal peer support service with targeted support for women living in specific areas of deprivation with low breastfeeding rates, identified by postcode. All women could call or text PSs for support, access online forums, or visit community support groups. Women living in target areas could sign up for proactive text and telephone support (for the first six weeks) when meeting PSs at antenatal classes. They could also be signposted by a health professional, or self-refer. Three PSs who were paid for a small number of hours per week but provided many more as volunteers, provided this early proactive support. Volunteer PSs attended antenatal classes and community groups.

### Recruitment

At each site, a range of stakeholders (mothers who had/had not engaged with peer support, PSs, BPS manager, health professionals, commissioners) were recruited to capture a spectrum of experiences from different standpoints. It was also intended that observations of peer support supervision sessions would take place at each site (but were not achieved at Site 1 because the BPS contract had been re-tendered. As another non-profit organisation was soon to take over, no further supervisions were planned).

Mothers were given information sheets and reply slips and recruited either by PSs, at health visitor clinics or community groups, or via snowball sampling. At both sites, all mother participants lived in areas of deprivation targeted by the service.

Health professionals, PS, non-profit managers and commissioners were recruited via information sheets sent via line managers to their work addresses.

### Data collection

Data collection took place during 2018. Interview schedules were designed for the different population groups, and all included questions on their experiences of the service. The key issues of mother’s engagement/non-engagement with the services and professional participants’ experiences of service development, strategies to engage with the target population, decision-making processes, and inter-professional collaboration were explored. Interviews took between 7 and 45 min, with a mean length of 26 min. A small number of interviews with non-engaged mothers were short (i.e. 7 min) because they had not heard about the service and had no experience of it. Interviews were audio recorded, transcribed and uploaded onto qualitative data analysis software (MAXQDA) for analysis. Detailed notes were taken during the PS supervision session observed at site 2 which took 90 min. All participants were able to request a copy of the main themes and after data analysis, were invited to take part in a member check interview.

### Data analysis

Data collection and analysis were concurrent with field notes taken immediately following interviews. Inductive analytic techniques developed via grounded theory methods outlined by Charmaz [42] were used. This included open coding to name and categorise the data, memos to record questions about codes or instances within the data, and constant comparisons (whereby instances within and between texts were compared and memos written about the comparisons) to ‘*establish analytic distinctions’* ([42], p.54). These analytic steps were iterative and continued until theoretical ideas emerged. Finally, cross case analysis was undertaken to identify similarities and differences between the cases [43]. Gradually, codes were grouped to form themes, and theoretical links between the themes were made. As analysis progressed, one theme theoretically underpinned the others and became the overarching theme. LH led on the analysis, with all theoretical ideas discussed and agreed amongst the authors.

Seventeen participants (8 women, 6 PSs, 1 manager, 1 health professional, and 1 commissioner) opted to take part in a member check interview during which broad agreement with the themes was expressed.

#### Findings

Overall, forty interviews (face to face (*n* = 7), telephone (*n* = 33)) were undertaken (see Table 1), 20 at each site, and one observation at site 2 (involving 10 participants).

**Table 1:** Interview Participants

Participant	Number interviewed	
	Site 1 (Organisation A)	Site 2 (Organisation B)
Peer supporters	4	5
Mothers who had engaged with service	5	5
Mothers who had not engaged with service	5	4
Peer support service manager/peer support co-ordinator	1	2
Community midwives	1	0
Health visitors	2	2
Infant Feeding Co-ordinator	1	1
Commissioner	1	1
Total	20	20

The socioeconomic characteristics of women and peer supporter participants are detailed in Table 2.

**Table 2:** Socio-economic characteristics of women and peer supporter participants

Role	Age	Postcode IMD Quintile	Education	Ethnicity	Infant feeding history
**Site 1 (Organisation A)**					
Peer supporter (S1PS1)	33	2	Degree	White British	Exclusive and continued breastfeeding
Peer Supporter (S1PS2)	40	2	Degree	White British	Exclusive and continued breastfeeding
Peer Supporter (S1PS3)	30	3	Degree	White British	Mixed feeding and continued breastfeeding
Peer Supporter (S1PS4)	49	5	Degree	White British	Exclusive and continued breastfeeding
Mother engaged with service (S1EM1)	34	1	Degree	White British	Exclusive breastfeeding and mixed feeding
Mother engaged with service (S1EM2)	35	1	Degree	White European	Exclusive and continued breastfeeding.
Mother engaged with service (S1EM3)	29	1	Education until age 18	White British	Exclusive and continued breastfeeding
Mother engaged with service (S1EM4)	28	1	Education until age 18	White British	Exclusive breastfeeding, mixed and formula feeding
Mother engaged with service (S1EM5)	23	1	Education until age 18	Asian British	Breast milk feeding and formula feeding
Mother not engaged with service (S1NEM1)	25	1	Education until age 18	White British	Formula feeding
Mother not engaged with service (S1NEM2)	23	1	Education until age 18	Asian British	Breast and formula feeding
Mother not engaged with service (S1NEM3)	39	1	Education until age 12	White British	Formula feeding
Mother not engaged with service (S1NEM4)	31	1	Education until age 18	White British	Exclusive and continued breastfeeding
Mother not engaged with service (S1NEM5)	21	1	Education until age 18	White British	First breastfeed. Formula feeding
**Site 2 (Organisation B)**					
Peer supporter (S2PS1)	43	4	Education until age 18	White British	Exclusive and continued breastfeeding
Peer Supporter (S2PS2)	38	3	Degree (PhD)	White British	Exclusive and continued breastfeeding
Peer Supporter (S2PS3)	31	4	Degree (PhD)	White British	Exclusive and continued breastfeeding
Peer Supporter (S2PS4)	29	3	Degree	White British	Exclusive and continued breastfeeding
Peer Supporter (S2PS5)	48	1	Degree (PhD)	White British	Exclusive and continued breastfeeding
Mother engaged with service (S2EM1)	23	2	Degree	White British	Exclusive breastfeeding
Mother engaged with service (S2EM2)	23	2	Degree	White British	Exclusive breastfeeding and mixed feeding.
Mother engaged with service (S2EM3)	36	3	Degree	White British	Exclusive and continued breastfeeding
Mother engaged with service (S2EM4)	37	3	Degree	Asian British	Exclusive breastfeeding and mixed feeding
Mother engaged with service (S2EM5)	35	3	Degree	White British	Mixed feeding
Mother not engaged with service (S2NEM1)	20	1	Education until age 18	White British	Exclusive and continued breastfeeding
Mother not engaged with service (S2NEM2)	25	2	Education until age 18	White British	Formula feeding
Mother not engaged with service (S2NEM3)	28	3	Education until age 18	White British	Exclusive breastfeeding and mixed feeding
Mother not engaged with service (S2NEM4)	35	2	Education until age 18	White British	Breastfeeding and formula feeding

The findings from this study are brought together as four main themes that underpin an overarching theme of **‘*the transcending influence of society’***. This overarching theme offers insights into the wider policy and cultural context that influenced service development. The four main themes explain how BPS services developed within this background. They illustrate how PSs were resourceful in adapting their services (*‘adapting and modifying the support’*). The second theme refers to how BPS services worked to enable women’s access to supportive breastfeeding environments, but did not necessarily focus service development on the needs of women living in areas of deprivation (*‘supporting women’s journeys to access’*). Theme three, *‘embedding within healthcare practice’* explains the BPS-professional connections for supporting access and how BPS could result in more supportive community environments. Finally, theme four highlights data use including how management practices did not aid focus on context led service development (*‘ways of using knowledge’*). Each quote has a corresponding identifier containing a pseudonym, site identifier (i.e. S1), participant group (i.e. NEM1; non-engaged mother number 1), and transcript line number (i.e. {21}).

The overarching theme, ***‘the transcending influence of society’*,** captures the relevance of policies and the wider cultural context to service development, and explains the conditions in which the findings represented in the other themes emerged. At both sites, commissions followed principles of proportionate universalism whereby universal services were delivered at an intensity and scale proportionate to need [44]. Data sharing policy interacted with these proportionate universal aims influencing the extent to which they could be achieved. For example, because data policy dictated the transfer of minimum information [45], at site 1, the hospital did not provide PSs with women’s postcodes at hospital discharge. PSs were therefore unable to target support towards women living in target postcodes at the crucial first contact opportunity. At site 2, despite year-long interprofessional working, a data sharing agreement allowing midwives to sign up target women for early text support was not agreed; reducing sign up opportunities for target women.

Service intensity and universality were governed by funding, which in turn influenced the ease with which PSs could learn about women’s contexts. At site 1, adequate funding and data sharing arrangements enabled PSs to provide an intensive service including home visits which, although costly, afforded otherwise unobtainable insight into women’s wider social context:

*‘It’s opened my eyes to [ … ] a lot more of the struggle that locally, mums are facing and there’s families that only live a few streets away from me, and I never knew how bad it was for them’* (Kerry S1PS1 {105}).

Site 1 PSs demonstrated in-depth knowledge of target women’s contexts; for example, recognising target women might be less likely to seek formal information, have family members unsupportive of breastfeeding, and have lower confidence resulting in reduced service access. They also recognised target women might be coping with wider issues such as food insecurity, responsibilities for caring for older family members, language barriers, struggles with literacy, and lack of social support.

At site 2, PSs face-to-face contact with women was limited to clinics or community groups which provided fewer opportunities to learn about target women’s wider contexts. Although the site 2 PS co-ordinator recognised the interpersonal contexts mentioned above, she and the other site 2 PSs demonstrated limited understanding of wider social contextual issues. Most site 2 PSs did not realise that many women living in target areas stopped breastfeeding early. They did not mention the need for early support, and wanted to establish more community groups which were usually only accessed once babies are six weeks or older. Being less aware of wider contextual barriers seemed in tension with assumptions underpinning the aims of the commission and highlighted a lack of appreciation of the contextual challenges that women may face.

At both sites, service development was also influenced by participant views about equality of access. Although PS’s views were the main focus, these ideas were also present in other participant’s accounts, for example, a public health commissioner, health visitors and women. PSs actively sought to avoid categorising women, and rejected differential responses based on social factors:

‘*We have the same approach with everybody [ … ] Yes, we’re a non-judgemental service’* (Penelope S2PS1 {163}).

PSs valued equality of opportunity, so that *‘all mothers [...] have the same opportunity for support, or not’* (Sarah S1PS2 {56}), whereby individual needs rather than a population-based focus formed a key driver for service delivery:

‘*We are measured on those target areas [...] but I think our general aim is to give mums universal support rather than making it any more different for one mum because she lives in one postcode compared to another, so it is just on the needs of those mums we speak to’* (Kerry S1PS1 {88}).

These cultural aspects of society (discomfort when socio-economic groupings were mentioned, valuing equality of opportunity, and preferring to think about individual needs), interacted with the policy environment (proportionate universal policy, data sharing policy, and funding levels emanating from policies to fund services) to privilege focus on the individual, and steer attention away from focus on context. This underlying cultural and political context impacted on service development as reflected in the following themes.

##### Theme 1

**‘Adapting and modifying the support’** describes how peer support was acceptable to women, and how in response to local conditions PSs demonstrated proactivity, adaptability and resourcefulness.

Mothers from both sites who had received peer support liked it. They appreciated the practical, emotional, affirmational, and informational support PSs delivered in a person-centred, non-judgemental, non-directive way. Mothers also appreciated PSs availability, and proactive contact was acceptable to all mothers who received it. For example, when Tracey was worried about her baby’s weight gain, her peer supporter demonstrated she was present and available by discussing the situation face to face, and following up with proactive online messages:

*‘But X* [peer supporter] *was really good she like went through the reason why and messaged me on Facebook and stuff like that’* (Tracey S1EM1 {159}).

Many women who had not engaged with peer support lacked the opportunity to do so, and most women interviewed who had not received the service appreciated the idea of peer support, especially valuing the idea of PSs experiential knowledge:

*‘If another mum’s experienced something like that and... she* [new mother] *could talk to somebody who’s actually gone through it, it’d be like better than speaking to a midwife that has... learnt it off, like, paper’* (Carrie S1NEM1{20}).

PSs at both sites were seen to be adaptable, performing different functions at different time points along a mother’s journey. This was initially through provision of ongoing one-to-one support, and then by expanding mother’s social networks via access to online or community groups, because:

*‘If they’re going to group regularly and they’re meeting other people who are breastfeeding it gives a bit of balance [...] that it can be normal to breastfeed a baby beyond, you know, up to six weeks’* (Ellen S1PS4 {62}).

Resourcefulness was a hallmark of PSs work. At site 1 PSs were able to offer proactive home visiting which provided opportunities for early intervention. PSs were able to help prevent breastfeeding complications through early support, and to signpost women into services whether related to baby feeding or not. For example, when Kerry visited a mother concerned about benefits, housing and food security, she explained, *‘that visit for me was about putting her in touch with other services’* (Kerry S1PS1 {95}).

At site 2, PSs communicated mainly via text and met women either at health clinics or community groups. They could not reach everyone. As the commission required them to meet quarterly targets for text support, and antenatal classes yielded inadequate numbers, a strategy of *‘piggy backing’* onto other post-natal services was employed. For example, PSs attended a midwifery drop-in clinic at the hospital and community health visitor weigh in sessions. This strategy facilitated contact, but meant PSs met mothers in environments controlled by health professionals, which mothers may attend because a difficulty had already arisen. Janine explained that her main concern was to help with the identified issue:

*‘Because I’m* *mainly* *doing the … like the more intensive supporting at the hospital* [Saturday morning midwifery clinic] *- it’s literally ‘let’s deal with your issues, let’s have a chat, let’s give you some support’* (Janine S2PS5 {176})*.*

The strategy of ‘piggy backing’ meant providing information early (thereby preventing issues arising) tended to form a smaller part of the role and dealing with difficulties a larger.

##### Theme 2

**‘Supporting women’s journeys to access’** explains how strategies to facilitate better access were developed and could become compromised. The impact of contextual factors on mothers’ access pathways are then discussed.

At both sites, development of pathways to enable service access was not commonly focussed on the needs and contexts of the target population. For example, at site 1 although PSs established which mothers lived in quintile one postcodes (by asking them during the initial 48 h phone call), their access pathway was developed by sending texts to all mothers when their babies were 2–3 weeks old:

*‘That’s the point where paternity leave tends to be over for a lot of partners, [...] women are suddenly on their own at home with a baby so [...] at the end of year 1, we added an extra text in at 2 – 3 weeks to say ‘this is where we are, this is how you contact us and this is where our local breastfeeding groups are”* (Jackie S1Manager {20}).

This development utilised resource at a time when target mothers might be more likely to have already stopped breastfeeding [7], and the underlying thinking was aligned with needs of more socially advantaged mothers; it assumed they would have a partner who had been able to take leave, and would access services in response to a text.

Special pathways for target women were sometimes set up, however. For example, when site 1 PSs *‘found we were losing’* (Sarah S1PS2{32}) many young mothers, they used data provided by the hospital about mothers’ ages to constructed a new pathway so that:

*‘The first person they* [young mothers] *chat to is the same person that’s going to come in the door, [...] it’s the same person that will follow them up for as long as they need, and we found that much better’* (Sarah S1PS2 {28}).

Nonetheless, such special pathways could become compromised. For example, when PSs at site 2 were first asked to target women living in particular geographical areas, they initially used paid PSs to work in the hospital signing up target mothers for ongoing text support post discharge. As this yielded inadequate numbers of mothers, the pathway was opened to all:

*‘What we found is we wasn’t getting enough mums from just the X* [target area]*, so we expanded it to the whole of X* [city]*’* (Penny S2PS Co-ordinator {163}).

Although all site 1 mother participants were socio-economically disadvantaged, at site 2, despite all mother participants living in target areas, some were more socially advantaged than others (see Table 2). While the small sample precludes any meaningful comparison, when considering the developments to facilitate access at both sites (discussed above), one interpretation is that social disadvantage systematically impacted access at a gradient so that more socially disadvantaged mothers were less likely to receive the resource. For example, at site 2, several socially disadvantaged participants missed the opportunity to sign up for early pro-active text support because they did not attend ante-natal classes. Classes did not appeal as women felt *‘not interested’* (Carrieann S2EM2{142}), or that *‘I don’t want to go’* (Cerys S2NEM1{29}). Meanwhile in the hospital environment, more socially disadvantaged women seemed to struggle to ask for help. Avisa, aged 23, lived in a quintile one area and received schooling until age 18 expressed:

*‘They do say if you need help you can, but it’s a bit nerve racking asking sometimes’* (Avisa S1NEM2 {8}).

Several site 1 mother participants either did not pick up their 48 h phone call, or for some reason did not receive it. Maggie, for whom English was a second language, did not like to answer the phone without her husband present to help her, and her husband had no time off work when their baby was born:

*‘My husband work* [s] *in the morning* [s] *so nobody can answer the phone’* (Maggie S1EM2 {57}).

Meanwhile, being single, caring for all their children alone, and having no transport (as was Kiera’s situation) could make mothers’ community group participation more difficult:

*‘I didn’t come back* [to group] *cos it was a bit of a while away from my house.....and my little girl finishes nursery at the same time as it starts so I didn’t get back to it’* (Kiera S1EM4 {48}).

This data does not prove more socially disadvantaged mothers were less likely to receive peer support than the more socially advantaged, but it demonstrates how diverse contextual issues operating in different locations and time points in a mother’s infant feeding journey affected mothers’ access to BPS in this study.

##### Theme 3

**‘Embedding within healthcare practice’** is linked to service access. It explains how PSs can become integrated within health professional practice and the community, resulting in cultural change.

In order to build relationships and ensure BPS was embedded into local health services, effective and ongoing communication was needed. At site 1, funding afforded senior PSs management time to attend infant feeding strategy meetings and to conduct regular formal communication with health teams about their service:

*‘Once every 6 months* [I] *go round the all the different health teams just to chat to them... talk about the service, [...]things that have gone well, things that we’re finding challenging ... and also trying to really encourage at our infant feeding meetings [...] having that open discussion’* (Ellen S1PS4 {132}).

Although some site 1 health professionals desired more communication with PSs, the BPS service was considered to be *‘embedded within their* [health professional] *tool kit’* (Sarah S1PS2{134}). At site 2, despite BPS representation at the infant feeding strategic partnership meetings, the restricted budget meant the co-ordinator had to work over her paid hours to try and fulfil management duties and support women; with inadequate time for systematic formal communication with health teams:

*‘We’ve got a contact number...- that I can’t remember the lady’s name but we’ve got the contact number and details* *we* *call if needed or signpost on to parents but no there’s no regular sort of meetings or calls’* (Suzie S2HV2 {287}).

The lack of opportunity to build relationships with health services meant that there were low levels of service awareness amongst health professionals and mothers. Some women *‘stumbled across’* (Jane S2EM3 {82}) the service by chance, while several non-engaged women who would have liked peer support, did not know it was available. One health professional had *‘no idea’* (Maria S2HV1 {20}) why some women received text support, while others did not. At site 2 mothers’ referral to PSs remained unusual because peer support was *‘not kind of built in to our daily role’* (Suzie S2HV2 {307}).

At both sites the peer support service was considered to have led to community level change, although it was recognised across the BPS services that cultural change was a long-term effort.

At site 1 some PSs reflected on how support provided to one woman had a ‘ripple effect’ across the community:

*‘When I’m visiting mums they might say ‘oh you visited my friend’ [ … ] because they’ve been successful at breastfeeding and they’ve overcome the issues [...] they’ve taken the information away that we’ve given them and passed it on to other mums, so it has a ripple effect, so you might see one mum, but that might affect three mums outside of that’* (Kerry S1PS1 {128}).

At site 2, in addition to such networking by mothers, PSs sought to embed themselves within target communities by attending various community groups under the premise of changing attitudes about breastfeeding:

*‘It’s very much about volunteers [ … ] being there [ … ] weekly in the local communities where mums are, [ … ] sort of giving mums that kind of … access to support, [ … ] just building friendships really and then from that, you think, ‘oh actually the peer supporter’s quite a nice person’, you know ‘I might try breastfeeding’. It’s kind of those drip drip drip bits of information’* (Penny S2PS COORD {63}).

##### Theme four

**‘Ways of using knowledge’** outlines how contextual knowledge lacked visibility within management practices and how formal data sources were viewed as necessary for commission fulfilment rather than as potential service development tools.

The lack of contextual knowledge within management practices (e.g., supervision) appeared to have made it difficult for services to develop to fit local contexts. Supervision focused on *‘quality assurance’* by *‘making sure that we’re all working to the same ethos and standards’* (Ellen S1PS4 {3}), to facilitate ongoing learning, and as a means to *‘look after their [PSs] own resilience and wellbeing’* (S2PS3 Nina {57}). Commissioners managed the BPS services via key performance indicators (KPIs), used qualitative data and informal discussion at infant feeding strategy meetings, and wanted to *‘really be sure’* services were meeting *‘local mums’ needs’* (Mary S2Commissioner {67}). However, there were no systems in place to monitor context led service development. Indeed, during member check interviews, Ellen, an experienced site 1 peer supporter, explained she felt that context led development was not necessarily expected, but could happen *‘inside my head’* (Ellen S1PS4 Member check interview).

Formal data sources were viewed as necessary for commission fulfilment, rather than service development tools:

*‘Yes, there’s X* [manager of project] *that writes the reports and she’s interested in where these mother’s live and how old they are [ … ] because [...]that’s what the commissioners asked for, but I think for the rest of us on the ground it doesn’t actually make any difference’* (Sarah S1PS2 {56}).

Peer support managers at both sites used data sources such as peer support activity logs, group attendance logs, supervision feedback, and at site 1, mother’s survey feedback, to report quarterly on various KPIs. Jackie explained:

*‘There was a huge number of, KPIs to report on [ … ] so, things like [ … ] the number of 48 hour calls we made to women in a quarter, number of home visits, number of home visits to under 20’s, number of home visits to quintile one post codes’* (Jackie S1 Manager {9}).

However, at both sites the potential of hard data to assist service development was not fully realised. For example, at site 2, although PSs collected postcodes from all women engaging with the service, they did not scrutinise these to establish which women were more likely to engage. Furthermore, when new developments designed to improve access were introduced, their impact on the access of target and non-target women was not evaluated at either site (see theme 2).

## Discussion

This study reports the development of two BPS services in areas of deprivation. Its main finding is that access to services was a key issue. Non-profit organisations sought to enable women’s access to individual, social and community environments supportive of breastfeeding, but developments to facilitate access were not always tailored to the needs of the target women. Context led service development was not the main focus.

The findings that women appreciated the practical, emotional, affirmational, and informational support that PSs provided is reflected in the wider literature (e.g. [16, 46]). The importance of access also links with studies of BPS implementation in similar populations in other high-income countries such as the USA, where developing services to enable women to access peer support in the very early post-partum period has been important [47], and where PSs have developed novel outreach strategies [48]. It also links to Trickey et al.’s [49] realist review which reported accessibility to peer support as a key issue [49]. Our study builds on this finding highlighting how access can link to the wider political context. In our study when pathways enabling women’s access to peer support were developed, protection of the individual (as exercised via data sharing law) took precedence over the needs of target women as a group. At site 1, PSs were provided with the names and phone numbers of all women discharged who were breastfeeding, but because UK data sharing law requires sharing of only the minimum personal data [45], this precluded sharing their postcodes. Therefore, at the first phone call, PSs did not know what proportion of the whole population was made up by target women and were prevented from specifically attempting to contact target women, and tailoring such attempts to women’s needs at this crucial early time point. Despite the policy intention (based on proportionate universalism requiring more resource reach target women [27]), data sharing law prohibited the kind of data sharing that would enable a proportionate universal approach. Studies discussing the impact of data sharing on equity of access have not been identified. However, public health researchers are urged to look to the political determinants of health inequalities in order to identify the actors and forces driving them [50]. The finding that the policy outworking of individualism (i.e. data sharing law) affected resource allocation, even when a proportional universalism policy had been adopted, provides an empirical example of how political ideology impacts on practices and outcomes.

The findings from this study emphasise the significance of contextual issues. For example, Trickey et al.’s [49] realist review viewed PSs’ practice of being mother focussed as a mechanism to enable mothers to keep breastfeeding [49]. Our findings hint at a more complex relationship between a mother focussed approach, women’s wider contexts, and breastfeeding practices; suggesting that, in the context of socioeconomic deprivation, when a peer supporter focuses on the needs of a mother, she may be working to help address wider contextual issues as well as affecting the mother’s own internal motivation to breastfeed. This juxtaposes to some degree with the findings of Copeland et al. [51], who, in their study, suggest the need for the PSs to maintain the focus of their conversations with mothers upon breastfeeding. Trickey et al. [49] found that PSs were more motivated when their work was appreciated, and more responsive to mothers who actively sought their support. Trickey et al. [49] suggest this may drive the trend for more socially confident and advantaged women to receive more peer support. Our findings expand upon this explanation, suggesting that access inequity may result from a combination of the barriers to access affecting more socially disadvantaged women, and the genuine desire of peer supporter’s and their organisations to help everybody without reference to context.

Improving the fit between BPS services and the needs of target women through context led service development was not a central focus of the services’ activities. Service decentralisation is the policy of delegating central government powers to local or regional authorities [52]. It theorises local actors, such as non-profit organisations, have special knowledge of communities because they are closer to them, more sensitive to local conditions, and better able to respond to local needs [53]. Despite scant evidence of such special knowledge [54], decentralisation has been used to justify the role of non-profit organisations in UK service delivery [31, 55–58]. In our study, although there were occasions when PSs used their contextual knowledge to inform the development of access pathways for target women (i.e., the young mothers’ pathway), PSs did not always recognise the value and relevance of mothers’ contexts (i.e., their wider family and community contexts, levels of deprivation and socio-economic situations in which they lived), and some service developments did not take mothers’ contexts into account. Further, organisational processes did not facilitate the acquisition and use of contextual knowledge, and identification of aspects of the context affecting target women, and service developments responding to such issues did not form part of commission reporting requirements. Hence, improving the fit between BPS services and the needs of target women did not form a central focus of the services’ activities. This finding calls for contextual knowledge to be made visible through the development of management practices that formally enable its capture and utilisation. Specifically, the social ecological approach [59] could be used to theoretically inform development of a practice tool whereby macro, meso and micro level influences are taken into consideration. The social ecological approach has been used to underpin calls for the creation of conditions conducive to generating change in global [60, 61] and national [62] breastfeeding rates, to theoretically structure analysis of a BPS service evaluation [63], and has been highlighted for its utility in linking individual experiences to wider social drivers within conversations about infant feeding [64]. Such a tool would require evaluation and testing, but could be utilised to guide discussion about context during supervision sessions, as part of commission feedback, and during service development decision making. This could benefit the women the services aim to target by making services more attuned to their needs and ensuring ongoing management focus upon them and the nature of their wider social contexts. The maintenance of focus on women’s wider contexts, including on influences affecting their service access could increase equity by helping ensure resources reach those living in deprivation. It also has the potential to increase equity indirectly by prompting non-profit organisations to review their advocacy role in society. This could form an example of evidence-based advocacy which is anticipated to help generate strengthened political will and commitment to creating conditions conducive to changes in breastfeeding rates [61].

In addition to the provision of individual support, findings suggest organisations sought to facilitate women’s access to supportive environments at a social and community level. This is in line with a large body of literature suggesting the negative impact of an absence of breastfeeding knowledge within the community and by social contacts e.g., [65, 66, 67]. Our findings suggest networking as a mechanism, whereby mothers who have used the services and PSs talk to mothers and other people in the community about breastfeeding and BPS services. The concept of informal networking as a mechanism underpinning change at social and community levels is not new [68], yet there are few theories that underpin community level BPS [49, 69]. Social capital theory, with its focus on the bonds and links between people, has previously been used to analyse a peer support service [70], and resonates with the concept of networking. Future services could maximise networking by recruiting PSs resident in target communities who have multiple local social links. This could increase equity and benefit women living in target areas by increasing the likelihood of them meeting a peer supporter.

### Strengths and limitations

This study’s findings are interpretations of the experiences of a small number of participants and cannot be generalised to other situations. However, the ideas, theories and explanations forming the theoretical generalisations may help guide future studies of similar interventions. The qualitative approach utilised allowed participant’s voices to be heard. Incorporating the views of women who have and have not engaged with peer support as well as those of PSs, health professionals, managers and commissioners is one of the study’s strengths. A few weeks before gaining ethical clearance, the site 1 contract was re-tendered and awarded to another non-profit organisation. Hence, data collection there was rapid with all but one interview conducted via telephone and no chance to observe supervision. Lack of funding for interpreters meant mothers who could not speak English were not recruited. While at site 2 no such potential participants were encountered, at site 1 this meant several women could not take part. It is possible that participants put forward views they felt were desirable. Future studies could use serial interviews to build trust and reduce the likelihood of this happening.

## Conclusion

This study reports on the development of BPS services in areas of deprivation. It suggests access to services was a key issue, and that context led service development was not a primary concern. Service development was shaped by the interaction of proportionate universal policies (prioritising population needs), and data sharing policy (prioritising individual needs). Although peer supporters worked resourcefully within this milieu to facilitate women’s access to services, they did not always focus service development on the needs of target women and management practices did not enable focus on context led service development. A social ecological approach to theoretically inform the development of a practice tool whereby macro, meso and micro level influences are taken into consideration, may help facilitate the capture and use of contextual knowledge.

## Data Availability

The datasets used and analysed during the current study are available from the corresponding author on reasonable request.

## Abbreviations

BPS: Breastfeeding peer support
KPI: Key performance indicator
PSs: Peer supporters
